# Modulation of anti-tumor lymphocyte function by neurotransmitter glutamate

**DOI:** 10.1186/2051-1426-2-S3-P38

**Published:** 2014-11-06

**Authors:** Thomas Hodo, Menaka Thounaojam, Anil Shanker

**Affiliations:** 1 Meharry Medical College School of Medicine, Nashville, TN, United States; 2 Meharry Medical College School of Medicine / Vanderbilt-Ingram Cancer Center, Nashville, TN, United States

## 

Most research to date pertaining to neural influence on immune response involves immunosuppression via the anti-inflammatory pathway. However, there is emerging evidence indicating that neurotransmitters have the ability to promote immune activation. We are investigating whether neurotransmitters can modulate and/or activate T cell function in situations where immunosuppression is prevalent such as in the tumor microenvironment. Published work suggests that glutamate, serotonin, dopamine, and Substance P trigger immune responses such as cytokine secretion, integrin expression, and chemotaxis. We saw that both CD4 and CD8 T cells express high surface protein levels of glutamate receptor AMPA iGluR3, which is able to import Ca^2+ ^and Na+. We also found that mGluR1 is significantly upregulated on lymphocytes upon activation. Our data further show that T cells in the tumor-draining LN and tumor-infiltrating lymphocytes have upregulated expression of iGluR3 and mGluR1. Treatment with glutamate or its receptor agonist augmented T cell proliferation following CD3-CD28-mediated TCR stimulation. Thus, modulation of glutamate receptor signaling can be useful for enhancing anti-tumor T cell immunity such as inhibition of AICD, enhancement of proliferation, and increased cytokine production [1,2]. Indeed, overactivation of lymphocytes in multiple sclerosis is closely tied to the overexpression of AMPA GluR3 on T cells [3]. Experiments are under way to dissect in an adoptive transfer set up whether glutamate-modulated immune effector function involves specific activation of anti-tumor lymphocytes to elicit cytolytic response that is needed to cause tumor cell death. Our findings will help identify novel neuro-immune modulators that may serve to enhance anti-tumor T cell response.
